# Amine-Aldehyde Chemical Conjugation on a Potassium Hydroxide-Treated Polystyrene ELISA Surface for Nanosensing an HIV-p24 Antigen

**DOI:** 10.1186/s11671-018-2848-z

**Published:** 2019-01-14

**Authors:** Cunzhen Wang, Thangavel Lakshmipriya, Subash C. B. Gopinath

**Affiliations:** 1 Department of Intensive Care Unit, Henan Provincial People’s Hospital, Zhengzhou City, 450000 Henan Province China; 2 Centre of Innovative Nanostructure and Nanodevices, Universiti Teknologi PETRONAS, 32610 Bandar Seri Iskandar, Perak Darul Ridzuan Malaysia; 3 Institute of Nano Electronic Engineering, Universiti Malaysia Perlis, 01000 Kangar, Perlis Malaysia; 4 School of Bioprocess Engineering, Universiti Malaysia Perlis, 02600 Arau, Perlis Malaysia

**Keywords:** Enzyme-linked immunosorbent assay, Human immunodeficiency virus, p24 antigen, Amine-aldehyde, Surface chemistry

## Abstract

The enzyme-linked immunosorbent assay (ELISA) has been widely used for disease surveillance and drug screening due to its relatively higher accuracy and sensitivity. Fine-tuning the ELISA is mandatory to elevate the specific detection of biomolecules at a lower abundance. Towards this end, higher molecular capture on the polystyrene (PS) ELISA surface is crucial for efficient detection, and it could be attained by immobilizing the molecules in the correct orientation. It is highly challenging to immobilize protein molecules in a well-aligned manner on an ELISA surface due to charge variations. We employed a 3-(aminopropyl) triethoxysilane (APTES)- and glutaraldehyde (GLU)-coupled PS surface chemical strategy to demonstrate the high performance with ELISA. A potassium hydroxide treatment followed by an equal ratio of 1% APTES and GLU attachment was found to be optimal, and a longer incubation with GLU favored maximum sensitivity. p24 is a vital early secreting antigen for diagnosing human immunodeficiency virus (HIV), and it has been used for efficient detection with the above chemistry. Three different procedures were followed, and they led to the improved detection of the HIV-p24 antigen at 1 nM, which is a 30-fold higher level compared to a conventional ELISA surface. The surface chemical functionalization shown here also displays a higher specificity with human serum and HIV-TAT. The above approach with the designed surface chemistry could also be recommended for disease diagnosis on other sensing surfaces involving the interaction of the probe and the analyte in heterogeneous test samples.

## Background

The detection of disease biomarkers and surface antigens on intact pathogens is necessary in the field of medical diagnosis to extend the human lifespan and to support healthier lives. Different detection systems are available to diagnose pathogens and life-threatening diseases, including cancers [1–4]. The range of probes and sensing strategies has been shown to identify several diseases [5, 6]. Among these outcomes, the enzyme-linked immunosorbent assay (ELISA) is a well-established strategy for detecting and diagnosing major diseases including HIV, and ELISA has been considered as a quality control test [7–10]. As an immunoassay, ELISA detects the antigen using the appropriate antibody. To fulfill this role, the antigen is immobilized on a polystyrene (PS) surface and then interacts with the partner antibody (primary antibody), followed by the host-specific antibody (secondary antibody) with the conjugated enzyme, which is allowed to bind to the primary antibody. Finally, these molecular interactions are monitored by a suitable substrate. Researchers have used different ELISA-based approaches, including the sandwich method with suitable poly- and monoclonal antibodies or aptamer-antibody combinations to improve the detections [11–14].

The sensitivity of an ELISA depends on the parameters such as the interaction between the antigen and the antibody, the temperature, the pH, and the efficiency of the antigen attachment on the PS surface. Among these factors, the immobilization of the antigen or antibody on the PS plate plays a crucial role in improving the limit of detection. Moreover, the limitation posed by the attachment and the nonuniform distribution of protein on the PS surface highly affects the assay sensitivity [5]. A higher quantity with the proper immobilization of protein aids in achieving the right orientation on the PS surface for possible improvements in detection. Different strategies have been employed to immobilize proteins properly on the PS surface. A protein or antibody has the ability to immobilize on the PS plate simply by chemical or physical adsorption or electrostatic interaction. Bora et al. [15] immobilized protein on the PS surface using photochemistry, and they found improvements of up to 1.5- to 2-fold higher compared to the untreated surface. In another study, the efficient immobilization of protein on the PS plate with a polymer called polyvinyl benzyl lactonoylamide (PVLA) was shown [16]. Polyethylene glycol (PEG)-based polymers have also provided for the efficient immobilization of biomolecules on sensing surfaces. Lakshmipriya et al. [17] developed an improved detection of clotting protein factor IX by immobilizing its thiolated oligomer along with two polymers (PEG-b-PAAc and N6-PEG) on a gold-coated surface.

On the ELISA plate surface, PS is composed of an aliphatic carbon chain with pendant benzene rings on each carbon, and it provides a hydrophobic surface. The carboxyl group on the PS surface binds the antigen or antibody through an electrostatic interaction with its exposed amine groups. However, this binding strategy has disadvantages, such as the lower binding of protein and irregular positioning of the molecules. The smaller-sized proteins and peptides with a lower number of amino acid compositions are especially difficult to bind on the PS surface due to the availability of fewer epitopes. Furthermore, it has been shown that if the molecules on the sensing substrate are crowded, then the distance among the probes is too disturbed to engage in a genuine interaction. For that reason, increasing the binding of a protein or antibody on the PS plate with the proper orientation improves the detection limit. In general, proteins are directly immobilized on the ELISA surface, and researchers are focused on improving protein immobilization on the ELISA surface to develop high-performance detection. In particular, the immobilization of protein by chemical functionalization on the PS surface has been observed by several researchers to improve this strategy. Amine-based chemical modification is effective at immobilizing different antibodies through the appropriate cross-linker. 3-(Aminopropyl) triethoxysilane (APTES) is commonly used to modify the sensing surface for use with amines. The antibody can immobilize on the amine-modified APTES surface through its COOH group. However, the immobilization of protein cannot be linked directly onto the amine surface like the antibody; in particular, smaller-sized proteins require a proper cross-linking agent. Here, we introduced a simple step immobilization for protein using a chemical modification with APTES and glutaraldehyde (GLU). We immobilized the proteins covalently on the amine-modified surface and linked using GLU. GLU is an organic compound with the formula CH_2_(CH_2_CHO)_2_, and it has been found to be one of the most effective protein cross-linking agents [4, 18–20]. It has two aldehyde groups at its ends; one end binds with the amine-modified ELISA surface, and the other end is free to bind the protein or antibody. In general, the immobilization of smaller-sized proteins or peptides on the ELISA surface is truly challenging due to the presence of fewer amines on its surface. With the chemical functionalization strategy, there is a possibility of using smaller-sized proteins or peptides to immobilize the material strongly on the ELISA PS plate. For our method, we used APTES-GLU as the linker; by using this modification, we can immobilize any types of antibodies, proteins, and peptides. The signal and sensitivity enhancements under this strategy are higher compared to other chemical strategies explored in the past [3, 4].

To demonstrate the advantage of these chemistries, we chose the detection of one of the major human immunodeficiency virus (HIV) proteins (p24), which is expressed at the earlier stage of HIV infection. The p24 antigen is made up of the viral core and is present at a higher level during the first week of infection. Therefore, it is ideal to generate a sensitive system using p24 for earlier HIV detection. Herein, the detection of p24 was generated using the abovementioned chemically modified ELISA surface. This chemical functionalization strategy has improved the p24 detection on the PS ELISA surface over several folds, and it can be recommended for detecting other important clinical biomarkers.

## Materials and Methods

### Reagents and Biomolecules

Recombinant HIV-p24 and Tat proteins and p24 antibody were purchased from Abcam (Malaysia). 3-(Aminopropyl) triethoxysilane (APTES), glutaraldehyde (GLU), and human serum were purchased from Sigma-Aldrich (USA). Anti-mouse-IgG-conjugated horseradish peroxidase (anti-mouse immunoglobulin HRP) was obtained from Thermo Scientific (USA). An ELISA plate was procured from Becton Dickinson (France). An ELISA 5X coating buffer was obtained from Biolegend (UK). Bovine serum albumin (BSA) and HRP substrate [3,3′,5,5′-tetramethylbenzidine (TMB)] were obtained from Promega (USA). The analytical ELISA reader was obtained from Fisher Scientific (Malaysia).

### Optimum APTES and GLU Levels for Functionalization on the PS Surface

The optimization of the APTES and GLU levels to use on the PS surface was performed with the targeted HIV p24 antigen. To decide the suitable concentrations of APTES and GLU, the ELISA surface was first activated by 1% potassium hydroxide for 10 min. Then, three different amounts of APTES (0.5, 1, and 2%) were allowed to bind on the ELISA surface, and they were incubated overnight at room temperature (RT). After that, two different amounts of GLU (1 and 2%) were applied to the amine-modified PS surface. At that point, HIV-p24 at a 250-nM concentration was allowed to bind on the GLU-modified surface, and the remaining GLU surface was blocked with 2% BSA. The p24 antibody was introduced at a 1:1000 dilution, and then anti-mouse IgG-HRP was allowed to bind to the p24 antibody. Finally, these interactions were monitored by adding the substrate (3,3′,5,5′-tetramethylbenzidine, TMB) for HRP. After the optimal amount of substrate (100 mM) was added, the absorbance was read with an ELISA reader at 405 nm. Control experiments were performed in the absence of target HIV-p24.

### Optimization of Aldehyde Linkage to Amine Group

To determine the suitable incubation time for GLU to link the amine-modified surfaces, two different incubation times were observed. After the ELISA PS surface was modified with APTES, the GLU was immobilized for 3 h and independently incubated overnight, and then 250 nM HIV-p24 antigen was immobilized on the GLU-modified surfaces. The remaining steps were followed as shown in the previous experiment.

### Comparative Detections Between HIV-p24 Antigen on the GLU-Modified Surface and the GLU Premixed-HIV-p24 Antigen Attachments

To detect the HIV-p24 antigen on the ELISA surface, we compared two different surface modification approaches using GLU. In the first one (method 1: amine-GLU-p24), the PS surface was initially modified by an amine using 2% APTES, 2% GLU was added to the surface to tether the plate surface with an aldehyde group, and then 100 nM HIV-p24 antigen was added. In the second approach (method 2: amine-GLU premixed p24), after the surface was modified by an amine, GLU-p24 (2% GLU was mixed with 100 nM of HIV-p24 antigen and maintained at RT for 30 min) was added. Finally, the detection was performed using the p24 antibody. The other steps were performed as explained above. The control experiments were performed in the absence of the target HIV-p24.

### Detection Limit: Conventional vs Chemically Modified ELISA

To check the limit of detection, different concentrations of p24 were titrated from 0.5 to 500 nM, and they were evaluated using the conventional methods and methods 1 and 2.

#### Conventional method

HIV-p24 antigen was initially diluted with 1% ELISA coating buffer, added to the ELISA surface, and maintained at 4 °C overnight, and then the remaining areas were blocked with 2% BSA for 1 h at RT. After that, a 1:1000 dilution of p24 antibody was added and incubated for 1 h, and then a 1:1000 dilution of anti-mouse-IgG-HRP was added to the ELISA surface and allowed to stand for 1 h. Finally, the HRP substrate (TMB) was added and measured at a wavelength of 405 nm using the ELISA reader.

#### Method 1

Different concentrations of HIV-p24 antigen were added to the APTES- and GLU-modified surfaces. After the antigen was immobilized, the remaining steps were followed as stated in the conventional ELISA. Washings were performed five times between each immobilization step. The absorbance was recorded at a 405-nm wavelength using the ELISA reader, and photographs were taken.

#### Method 2

On the APTES-modified ELISA plate, different concentrations of p24 premixed with GLU were immobilized. All the other steps used for the conventional ELISA were followed. Washings were performed five times between each immobilization step. The absorbance was recorded at a wavelength of 405 nm using the ELISA reader, and photographs were recorded. Control experiments were performed in the absence of the target HIV-p24.

### Specific Detection of the HIV-p24 Antigen on the APTES-Modified Surface

To check the assay specificity, we performed two different control experiments. Instead of the HIV-p24 antigen, we used human serum or HIV-TAT protein premixed with 1% GLU and added them to the APTES-modified surface. All the other steps were followed as explained above. Control experiments were performed in the absence of target HIV-p24.

## Results and Discussion

**Fig. 1:**
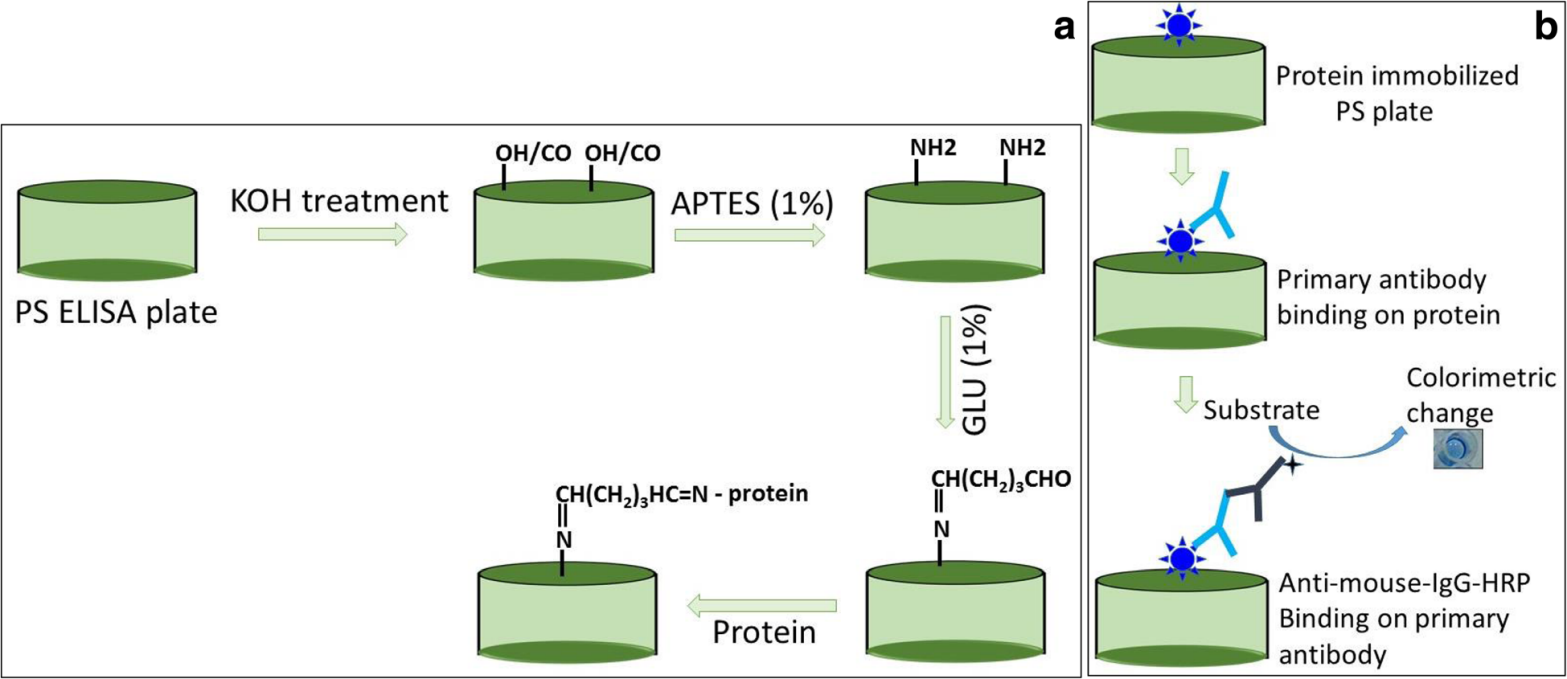
Schematic representation of chemically modified ELISA (**a**). The ELISA surface was modified chemically using APTES and GLU after potassium hydroxide treatment. The antigen was immobilized on the GLU-modified surface. **b** The immobilized antigen was detected by its partner antibody

The enzyme-linked immunosorbent assay (ELISA) is an effective immunoassay that helps researchers to identify different biomolecules using appropriate antibodies. A high immobilization of analytes (proteins, peptides, antibodies, and aptamers) [21–23] and the ability to capture molecules (monoclonal antibodies, polyclonal antibodies, and Fc regions of antibodies) [24, 25] can dramatically improve the limit of detection and assist in supporting the specificity of the analysis. With this in mind, several types of research are focused on chemically modifying the ELISA surface to capture the properly oriented probe or analyte at a higher quantity. In general, an antibody or protein is immobilized on the ELISA PS surface through an electrostatic interaction between the carboxyl group on the PS and the amine groups on the protein or antibody. However, this method is not efficient enough to attain a higher sensitivity and specificity due to its binding limitation. To overcome this fundamental issue, during the present investigation, we prepared a chemically functionalized ELISA surface using 3-(aminopropyl) triethoxysilane (APTES) and glutaraldehyde (GLU) for efficient protein immobilization. As shown in Fig. 1a, a potassium hydroxide-activated PS surface was modified by an amine using APTES and then cross-linked with GLU to attach the protein. In the absence of a potassium hydroxide treatment, the optimal level of APTES attached to the PS surface is significantly lower owing to the absence of a polar hydrogen bond acceptor that enhances the initial adsorption and will trigger the higher binding of APTES. Figure 1b shows the proposed detection of immobilized protein on the chemically modified ELISA surface with its partner antibody.

**Fig. 2:**
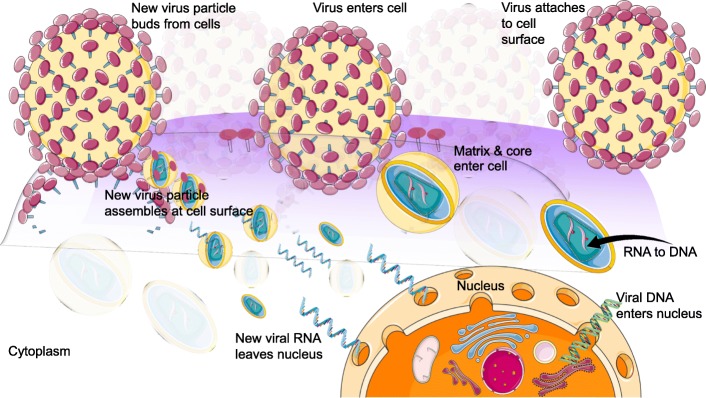
HIV and host cell interaction. The general strategy of HIV multiplication is shown

**Fig. 3:**
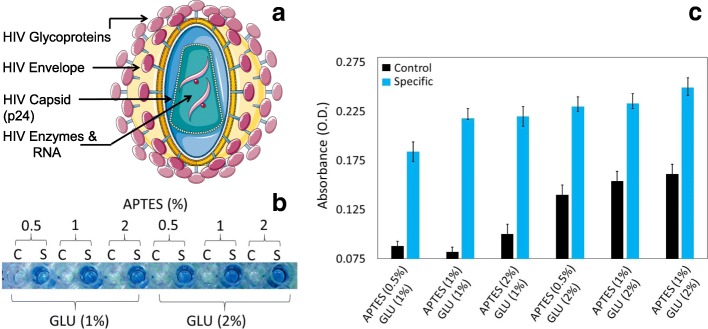
**a** Intact HIV structure. The p24 antigen is indicated. **b** The optimization of APTES and GLU. APTES (0.5, 1, and 2%) and GLU (1 and 2%) were used with different combinations to detect the constant concentration (250 nM) of HIV-p24 antigen

To collect evidence about this detection strategy, we wanted to use an important protein from the human immunodeficiency virus (HIV) called p24. The HIV-p24 antigen is one of the proteins that could be expressed during the early stage of HIV infection, and it multiplies in the host system as revealed in other viral systems (Fig. 2). Intact HIV has glycoproteins on the envelope covering the capsid, and the HIV-p24 antigen resides in the capsid region (Fig. 3a). Before detecting HIV-p24 antigen, we initially optimized the concentration of the chemical linker to capture the HIV-p24 antigen on the ELISA surface. We explored different concentrations and combinations of APTES and GLU on the ELISA surface and evaluated the results using a constant concentration of 250 nM HIV-p24 antigen. As shown in Fig. 3b and c, APTES and GLU application at 1% showed a saturated level of HIV-p24 antigen binding. If APTES was present a less than 1%, the binding absorbance (OD) was lower, at the same time level as that of 2% APTES, because of an increase in nonspecificity (in reference to the control experiment). For GLU, the 2% level shows good detection of the HIV-p24 antigen at an absorbance of 0.25, and simultaneously, the control experiment (without HIV-p24) displays the highest absorbance (0.16). The optimum 1% APTES and GLU show the lowest absorbance in the control experiment (0.08) and the highest absorbance (0.22) during the specific detection of the HIV-p24 antigen. This result occurred because, with the increase in APTES and GLU, it is possible to capture the antibody on the remaining free amine surfaces (originating from APTES) and aldehyde surfaces (originated from GLU). This result is the same even when the free surface regions were blocked with BSA in the above cases. To fine-tune the method further, we also made an attempt to increase the BSA concentration to minimize the background signal. However, even at the higher APTES and GLU concentration, there was biofouling. Based on these results, the optimized condition is 1% APTES and GLU, as used in the experiments.

**Fig. 4:**
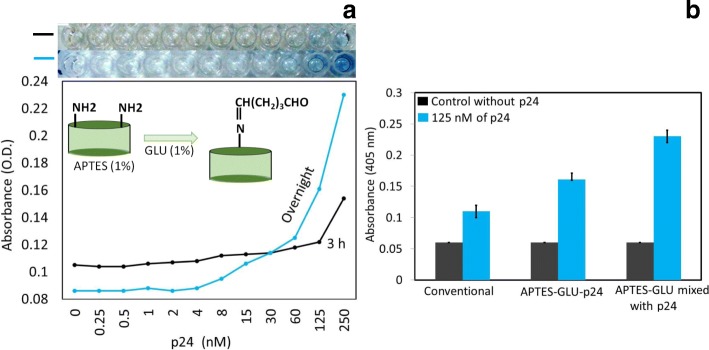
Optimization of the incubation period for GLU. **a** Optimized to have an incubation period of 3 h and an overnight incubation using 1% GLU on a 1% APTES-modified surface. **b** Detection of 125 nM p24 by three different approaches, conventional, APTES-GLU-p24 (method 1), and APTES-GLU-premixed p24 (method 2)

After the APTES and GLU optimization, we also adjusted the incubation time for linking the GLU onto the APTES-modified surface. As shown in Fig. 4a, incubating the GLU overnight leads to the highest absorbance compared with the shorter incubation time (3 h). This result occurs because of the insufficient incubation time for GLU to link to the APTES-modified surface; ultimately, the antibody is able to bind the remaining APTES surfaces. With an increased incubation time for GLU, this compound has a chance to cover the APTES surface completely. This saturation increases the protein immobilization on the GLU surface and enhances the assay sensitivity. At the 250-nM HIV-p24 concentration, there is almost twofold higher absorbance with the overnight incubation of GLU (Fig. 4a).

Due to the higher genuine signal following the overnight incubation of GLU with the increased absorbance and specific detection of p24, we used a similar condition for further experiments. In this case, we immobilized the GLU on the APTES-modified surface and then attached the protein (method 1). We also tried another approach; first, we mixed 1% GLU with 125 nM HIV-p24 antigen and then added this mixture to the amine-modified PS surface (method 2). Surprisingly, there was an increased absorbance with the same concentration of HIV-p24 antigen (from OD 0.161 to 0.23) as the concentration used in method 2. When we allowed the protein to bind to the GLU as the premix, almost all the proteins were able to bind the GLU, and then this mixture easily adsorbed onto the amine-modified surface. However, when the protein was allowed to bind to the pre-immobilized GLU surface, most of the aldehyde groups of the GLU were not available. Figure 4b indicates that the mixing method shows the detection of a similar concentration (125 nM) of HIV-p24 antigen with a higher absorbance. Additionally, methods 1 and 2 displayed higher absorbance compared with the conventional method under the same concentration of HIV-p24 antigen.

**Fig. 5:**
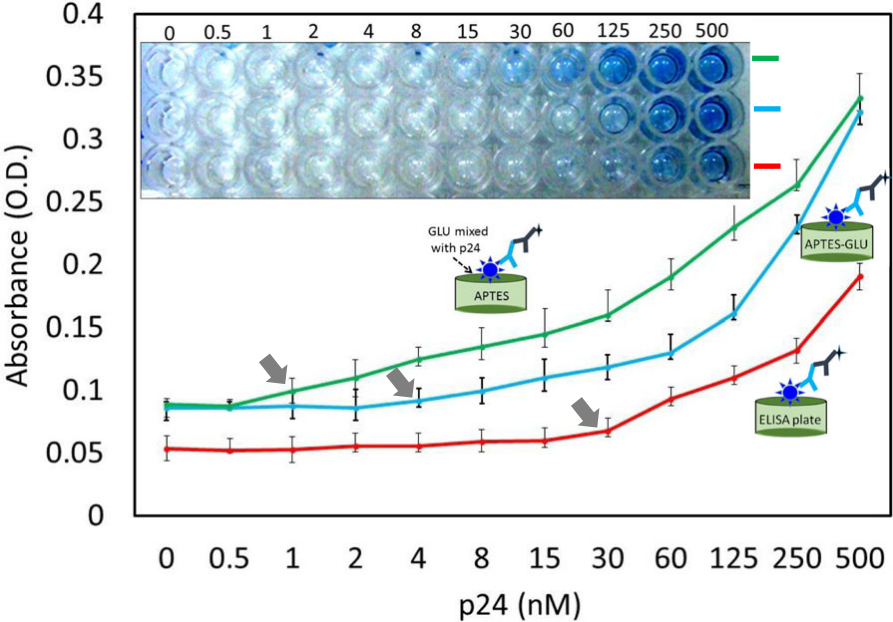
Limit of detection for the HIV-p24 antigen. The p24 was titrated from 0.5 to 500 nM. Three different approaches were followed, conventional and methods 1 and 2

After the complete optimization of the detection steps, to find the limit of detection, we titrated HIV-p24 antigen from picomolar to nanomolar ranges (500 pM to 500 nM). We compared three different methods, namely, the conventional, APTES-GLU-p24 (method 1: p24 immobilized on APTES followed by GLU modification), and APTES-GLU-premixed HIV-p24 (method 2: the premix of p24 and GLU followed by immobilization on APTES) with the same concentration of p24. As shown in Fig. 5, the limit of detection for p24 was found to be 30 nM for the conventional ELISA. In method 1, the limit of detection was improved eight times (4 nM) compared with the conventional ELISA. This result occurred because when we used the chemically modified PS surface, the protein immobilization was stable under the uniform arrangement and the immobilization rate was also quite high compared to the conventional adsorption of proteins on the ELISA surface. Vashist et al. [12] already showed that the higher immobilization of an antibody on the APTES-modified ELISA surface was associated with a dramatically improved detection level compared with conventional ELISA [12]. In their work, the amine in APTES can bind carboxyl groups on the antibody, and in that way, they immobilized the antibody chemically on the ELISA surface. Using this method, we can only immobilize the antibodies on the ELISA surface through its carboxyl groups, but protein-based antigen immobilization is not possible on APTES. For that purpose, we introduced the GLU linker to immobilize the protein on the ELISA PS surface. With these chemical linkers, not only protein but also an antibody was chemically linked through amine coupling to the aldehyde on the glutaraldehyde. The nonspecific attachment of biomolecules on the ELISA substrate was monitored using the control experiments. A control experiment was performed without the target molecule (HIV-p24). Without the target, the specific antibody for p24 cannot bind on the surface, and thus, the enzyme-conjugated secondary antibody is also not immobilized on the ELISA surface. In this case, when we added the substrate (TMB), we are not able to find any changes in the absorbance.

To improve the limit of detection, we tried to premix GLU and the HIV-p24 antigen before the immobilization step on the APTES-modified surface. It was expected that the premixture of GLU and HIV-p24 antigen would improve with the greater immobilization of proteins on the ELISA surface. When we premix GLU with the HIV-p24 antigen, the binding ratio of aldehyde to GLU is high. When we add this mixture to the ELISA surface, it improves the immobilization rate. Dixit et al. found that when they premixed the antibody and APTES prior to immobilization on the ELISA plate, their approach enhanced the immobilization of the antibody on the ELISA surface and the limit of detection was increased [12]. In our study, as expected, when we premixed the GLU and HIV-p24 antigen prior to immobilization, the limit of detection was improved to 1 nM and was 30-fold higher than that of the conventional ELISA, which showed a detection limit of 30 nM. Our strategy not only had an improved detection limit, but it also improved the absorbance for all the concentrations of HIV-p24 antigen. At 250 nM p24, we observed that the absorbance was 0.35, which is almost twice that of the conventional ELISA with the same concentration of HIV-p24 antigen. With all the HIV-p24 antigen concentrations, a large difference in absorbance was noted compared to the conventional ELISA (Fig. 5), as evident in the trustworthy detection of the HIV-p24 antigen.

**Fig. 6:**
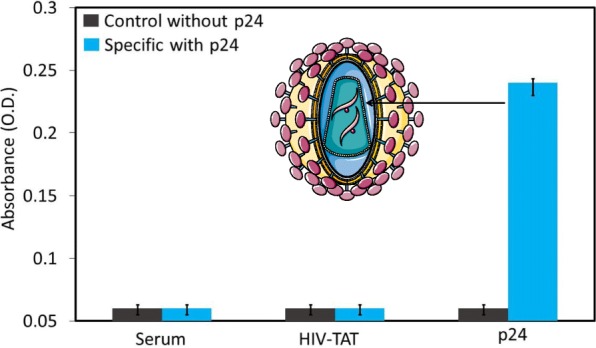
Specific detection of the HIV-p24 antigen. Instead of HIV-p24, the human serum and HIV-TAT protein were used to check the specificity

To evaluate the assay specificity, we performed the test with two different control experiments. We chose human serum and HIV-TAT and mixed with GLU instead of HIV-p24 antigen prior to use, and we evaluated their specificity. As shown in Fig. 6, in the case of human serum and HIV-TAT, there is no significant absorbance; however, 250 nM HIV-p24 antigen displayed a clear increase in absorbance. This result confirms the specific detection of the HIV-p24 antigen on the chemically modified ELISA surface with a higher sensitivity.

## Conclusions

A higher and proper immobilization of a protein or antibody on the ELISA surface dramatically improves the limit of detection. Here, we have introduced an interesting chemical functionalization method to immobilize the quantity of protein or antibody that binds on the ELISA PS surface, as assisted by APTES and GLU. To demonstrate detection, we used the p24 antigen from HIV. The limit of detection was improved by 30-fold compared with conventional ELISA. Further developments from the present investigation could lead to similar chemical-based improvements on other sensing surfaces and would be useful for detecting different antigens at a lower abundance, which would represent an advance in medical diagnoses.

## Abbreviations

APTES: 3-(Aminopropyl) triethoxysilane
BSA: Bovine serum albumin
COOH: Carboxyl
ELISA: Enzyme-linked immunosorbent assay
Fc: Fragment crystallizable
GLU: Glutaraldehyde
HIV: Human immunodeficiency virus
HRP: Horseradish peroxidase
IgG: Immunoglobulin
OD: One optical density
PEG: Polyethylene glycol
PS: Polystyrene
PVLA: Polyvinyl benzyl lactonoylamide
TMB: 3,3′,5,5′-Tetramethylbenzidine
